# Pathogenicity and Immunogenicity of a Serially Passaged Attenuated Genotype 2c Porcine Epidemic Diarrhea Virus Cultured in Suspended Vero Cells

**DOI:** 10.3389/fmicb.2022.864377

**Published:** 2022-04-12

**Authors:** Fei-Fei Ge, Long-Shan Kang, Li-Ping Shen, Hai-Xiao Shen, De-Quan Yang, Xin Li, Hou-Bin Ju, Hong-jin Zhao, Jian Wang

**Affiliations:** ^1^Shanghai Animal Disease Control Center, Shanghai, China; ^2^College of Life Sciences and Food Engineering, Hebei University of Engineering, Handan, China

**Keywords:** G2c-subtype strain, PEDV, pathogenicity, immunogenicity, different passage

## Abstract

In this study, one G2c-subtype strain of porcine epidemic diarrhea virus (PEDV) (SHXX1902 strain) was isolated from clinical samples in suspended Vero cells, which was different from the genotype of the commercial AJ1102 vaccine. As a result, we determined the pathogenicity of different passages’ isolates (SHXX1902 strain) and compared the immunogenicity of G2c-subtype strain (SHXX1902 strain) with the commercial AJ1102 vaccine. The viral titer reached 10^7^ 50% tissue culture infectious dose (TCID_50_)/ml, which met the requirement for seed virus replication during vaccine development. Five-day-old piglets were orally infected with viruses from passages P5 and P35 to determine the pathogenicity and immunogenicity of different passages. Pregnant sows were immunized with inactivated SHXX1902-P5 or the commercial AJ1102 vaccine (first immunized with an attenuated vaccine and then boosted with an inactivated vaccine) to study the influence of the culture method on the immunogenicity of the strain. The median pig diarrhea dose (PDD_50_) and the median lethal dose (LD_50_) of the P5 virus were 10^2.00^ and 10^2.84^ TCID_50_/ml, respectively. All five piglets infected with the SHXX1902-P5 virus shed the virus 24 h after vaccination, whereas only two of the five piglets treated with the SHXX1902-P35 virus shed the virus 48 h after vaccination. The SHXX1902-P35 virus was partially attenuated in the 5-day-old piglets. Inactivated SHXX1902-P5 induced PEDV-specific immunoglobulin G (IgG) antibody responses equivalent to those induced by AJ1102 after infection in sow serum. However, the IgA titer induced by AJ1102 was much higher than that induced by inactivated SHXX1902-P5 since the boost immunization. On days 5 and 7 after farrowing, the IgA titers were similar among the immunized groups. Our study highlights that serial passage can lead to the attenuation of G2c-subtype strain. The immunogenicity of the inactivated strain was similar to the commercial vaccine. Our observation helped conceptualize appropriate study designs for the PEDV vaccine.

## Introduction

Porcine epidemic diarrhea virus (PEDV) belongs to the order Nidovirales, family Coronaviridae, and genus Alphacoronavirus. PEDV is now a global pathogen. Mortality due to viral infection varies with age and has approached 100% in 1–3-day-old piglets. Its genome is arranged in the following order: 5′-untranslated region (UTR)-ORF1a/ORF1b-S-ORF3-E-M-N-3′-UTR (Oh et al., 2014). At present, two genotypes of PEDV are recognized. Each of which includes two subgenotypes. The subgenotypes of genogroup 1, which are weakly pathogenic, are classical G1a and recombinant G1b. The subgenotypes of genogroup 2, which are strongly pathogenic, are local epidemic G2a global epidemic or pandemic G2b and G2c (Lee et al., 2010; Lee and Lee, 2014; Oh et al., 2014; Lee, 2015).

There is worldwide porcine epidemic diarrhea (PED), first recorded in Britain and Belgium in 1971 (Wood, 1977). In the 1970s, PED first appeared in the United Kingdom and was caused by the G1a subtype of PEDV. It then spread to Europe and many Asian countries. Researchers developed a vaccine based on this subtype, which is widely used. In the past 30 years, the disease has only been sporadic. Since 2000, in South Korea, the G2a subtype has continued to be prevalent in pigs immunized with an anti-Gla-subtype vaccine. At present, the epidemic area of the G2a subtype is relatively limited and is mainly concentrated in South Korea, Thailand, Japan, and other Asian countries. In 2010, a PED outbreak caused by a G2b-subtype mutant strain was reported in China, which could have been caused by gene deletion, insertion, or point mutation in a virulent Gla-subtype strain. In April 2013, PED caused by a G2b-subtype virus broke out in the United States and spread rapidly to 33 states (Huang et al., 2013). Paarlberg (2014) conducted a loss assessment of this PED outbreak, confirming its damage to the United States pig industry.

The number of live pigs sold at the market decreased by 3%, and the losses to pig farmers and consumers reached US$1 billion (Paarlberg, 2014). At present, variant G2b strains are prevalent throughout the world. In 2014, an epidemic of a G1b-subtype virus was reported in the United States. The new G1b-subtype virus is different from the G2-subtype virus, with deletions and insertions in the S gene (Wang et al., 2014). The G1b subtype may have been produced by the recombination of a classical strain (G1a subtype) and an epidemic strain (G2 subtype) during the process of viral subgenomic transcription (Lee, 2015).

Moreover, reports of the newly discovered G2c strains increased significantly after 2012 in Europe. PEDV strains from different subgroups were also prevalent within the same areas, implying that the coincident “hot spots” in PEDV-endemic areas (e.g., China and South Korea) are critical in determining the sources of some PEDV variations. These hotspot areas are potentially important reservoirs for the evolution of genetic variation in PEDV, arising from recombination between the different PEDV subgroups (Guo et al., 2019).

The vaccination of sows during epidemics or outbreaks of PED is the basic method used to control and eliminate PEDV. Anti-PEDV vaccines include inactivated vaccines, weak viral vaccines, and other vaccines. Piglets can receive protection from the antibodies contained in the colostrum or milk from immunized postnatal females, thereby reducing the incidence of PED. Although PEDV was first detected in European pig populations, researchers did not develop vaccines because the disease was rare. However, in 1980, PEDV was detected in Asian pig populations, which became prevalent in many pig-raising countries. Therefore, weak and inactivated vaccines for G1-subtype strains (CV777 and SM98) were developed and widely used. In 2010, PED caused by a highly pathogenic G2b-subtype PEDV mutant broke out in many provinces in southern China, and the G1a-subtype vaccines did not provide effective immune protection against this mutant.

The difficulties of culturing G2c variant strains of PEDV and the low titers generated exacerbate the urgent need to develop highly effective vaccines against the prevailing PEDV strains. This study generated a serially passaged vaccine candidate (SHXX1902) with the short-term passage of a virulent PEDV isolate in suspended Vero cells. Compared with the parental strain, after passage 5 (P5), the cell-adapted strain showed deletions of 24 nt (equivalent to eight amino acids) and 52 nt (133 amino acids) in ORF1a and ORF3, respectively. Strain SHXX 1902-P5 shared 93.4–100.0% amino acid sequence identity in different regions with SHXX1902-P35 (Ge et al., 2021). Pregnant sows were administered the inactivated SHXX1902-P5 vaccine in two doses at a fortnightly interval before parturition. IgG antibody responses to PEDV were detected in the serum of sows until 2 weeks post farrowing. IgA antibody responses to PEDV were detected in the milk of all the immunized sows with inactivated SHXX1902-P5 vaccine on days 5 and 7 after farrowing. These results demonstrate that intramuscularly injected inactivated PEDV can be used as a vaccine in maternal vaccination strategies to provide durable lactogenic immunity and confer passive protection on litters against PEDV G2c.

## Materials and Methods

### Viruses

The isolation and continuous PEDV strain SHXX1902 in Vero cells have been reported previously (Ge et al., 2021). The fifth passage (P5) and 35th passage (P35) of SHXX1902 were used as virulent inocula, and their virulence was compared. Whole-genomic sequence analysis of these strains has been reported previously.

### Growth Kinetics

Vero cell monolayers were infected separately with PEDV P5 or P35 at a multiplicity of infection (MOI) of 0.01, or the cells were mock-infected in six-well plates. The culture supernatants and cell lysates were collected at 12, 24, 36, 48, 60, and 72 h post-infection. After one round of freeze-thawing, the cell culture samples were analyzed in 96-well plates with the Reed–Muench method to determine the 50% tissue culture infectious dose (TCID_50_/ml).

### Immunofluorescence Assay

Vero cells were infected with PEDV in 24-well plates at an MOI of 0.01. At 12 h post-infection (hpi), the cells were washed three times with phosphate-buffered saline (PBS), fixed with 4% paraformaldehyde at room temperature (RT) for 15 min, and then permeabilized with 0.2% Triton X-100 (Solarbio, Beijing, China) for 15 min. After the cells were washed as described above, they were blocked with 1% bovine serum albumin (Solarbio, Beijing, China) for 30 min at RT. A mouse anti-PEDV-S monoclonal antibody (Veterinary Medical Research and Development, United States) and Alexa-green- conjugated goat anti-mouse antibody were then used as the primary and secondary antibodies, respectively. The cell nuclei were counterstained with 4′,6-diamidino-2-phenylindole (DAPI; Vector Labs, United States) for 5 min at RT in the dark. The cells were washed three times with PBS and observed under a fluorescence microscope.

### Virulence Test of Subcultured Strains

The virulence of strain SHXX1902-P5 was studied in 5-day-old piglets. A virus solution (10^7^ TCID_50_/ml) was fed orally to the piglets at a 1 ml/piglet dose. Each group (groups 1–5) of five piglets was challenged orally with the 10-fold serially diluted (10^–3^–10^–7^) SHXX1902-P5 virus, whereas the control animals in group 6 were fed cell culture medium. Clinical symptoms, such as vomiting and diarrhea, were checked every day, and fecal swabs of the piglets were collected for PEDV detection. After 5 days, the piglets were killed, and intestinal samples were collected for pathological examination. Histological examinations were performed to determine whether SHXX1902 damaged the intestinal tracts of the infected piglets. Stool samples from the piglets in all groups were collected before infection and after that with 16-in. cotton-tipped swabs and scored for fecal consistency for 5 days after infection: 0 = normal, 1 = pasty, 2 = semiliquid, and 3 = liquid or watery. The median pig diarrhea dose (PDD_50_) and the median lethal dose (LD_50_) of the virus were determined as the reciprocal of the viral dilution at which 50% of the pigs developed watery diarrhea (score 3) or died, respectively, at a given time point, using the Reed–Muench method.

### Comparative Pathogenicity of Strains SHXX1902-P5 and SHXX1902-P35

The piglets were randomly assigned to one of three experimental groups: the SHXX1902-P5-infected group (*n* = 4), the SHXX1902-P35-infected group (*n* = 4), and the sham-infected control group (*n* = 4). The animals were fed commercial milk replacer four times daily. After a 2-day acclimation period, the piglets (5 days old) in the virus-infected groups were orally administered a 1-ml dose of one 10^5.84^ TCID_50_/ml virus (equivalent to 1,000 LD_50_, as determined in this study). The sham-infected pigs were administered a cell culture medium as a placebo. The animals were monitored daily for clinical signs of vomiting, diarrhea, and mortality throughout the experiment. Rectal swabs were collected from all the piglets, diluted with PBS to 10% (*w*/*v*) suspensions, and centrifuged. The clarified supernatants were subjected to real-time PCR with the RT-PCR Detection Kit (Weiboxin Biotechnology, Guangzhou, China) to detect PEDV shedding. The clinical significance score (CSS) was used to measure diarrheal severity with the following scoring criteria, based on visual examination for 7 days post-infection (dpi): 0 = normal and no diarrhea [mean cycle threshold (*Ct*) values of >45]; 1 = mild and fluidic feces; 2 = moderate watery diarrhea; 3 = severe watery and projectile diarrhea (mean *Ct* values of <20); and 4 = death. Throughout the study, the piglets were necropsied upon death after the challenge, whereas all the piglets that survived the challenge and the control groups were euthanized at 7 dpi for post-mortem examination.

### Pregnant Swine Vaccination Experiments

Vaccination experiments were conducted in six commercial crossbred pregnant sows (Great Yorkshire × Dutch Landrace) with the same parity and expected farrowing date. The animals were allocated randomly to one of three experimental groups: inactivated SHXX1902-P5-vaccinated group 1 (*n* = 3), group 2, treated with a commercial AJ1102 vaccine (*n* = 3), and a strict negative control group 3 (*n* = 1). A multiple-dose PEDV homologous vaccination schedule at bi-weekly intervals, starting before farrowing, was followed in this vaccination study. As the commercial vaccine was immunized according to the instructions, our isolates were not attenuated completely and were inactivated to immunize pigs. The three sows in group 1 were intramuscularly administered twice with a 1-ml dose of SHXX1902-P5 (10^4.5^ TCID_50_/ml) at 35 and 21 days prepartum. The three sows in group 2 were vaccinated with an attenuated vaccine and an inactivated vaccine AJ1102 (Wuhan Keqian Biology Co., Ltd., Wuhan, China). The strict negative control group 3 (*n* = 1) was used. All the sows were monitored daily for clinical changes and adverse effects following vaccination. Blood samples were collected from the vaccinated sows on days 0, 7, 14, 21, 28, 35, and 49 after the first immunization. Colostrum was collected, and milk was collected on days 3, 5, and 7 after farrowing.

### Reverse Transcriptase-Quantitative Real-Time PCR

Reverse transcriptase-quantitative real-time PCR (RT-qPCR) was performed with the One Step PrimeScript RT-PCR Kit (TaKaRa, Japan). A PEDV isolate with a known infectious titer was serially diluted 10-fold to generate a standard curve for each PCR. The samples’ viral concentrations (TCID_50_/mL) were calculated based on the standard curve. The mean *Ct* values were calculated for the PCR-positive samples, and the mean viral titers were calculated based on all the piglets within each group.

### Histopathology of the Small Intestines

Intestinal tissues and other major organs were grossly examined upon necropsy. Small intestinal tissue specimens (<3 mm thick) collected from each piglet were fixed in 10% formalin for 24 h at RT and embedded in paraffin according to standard laboratory procedures. The fixed samples were sent to Guangzhou Saville for histopathology.

### Porcine Epidemic Diarrhea Virus Enzyme-Linked Immunosorbent Assay

The sera’s PEDV-specific immunoglobulin G (IgG) was detected with ID Screen^®^ PEDV Indirect (IDvet, France). According to the supplier’s protocol, the PEDV-specific IgA in sera and milk was detected with a PEDV IgA ELISA antibody kit (Anheal Diagnostic, Beijing, China).

### Porcine Epidemic Diarrhea Virus-Neutralizing Antibody Detection

The sera and milk samples were serially diluted. Then, 50 μl of PEDV strain SHXX1902-P5 (2.0 × 10^3^ TCID_50_/ml) was added to an equal volume of the sera and milk samples. The mixture was then incubated for 1 h at 37°C and inoculated to a 96-well plate containing confluent Vero cells. After 2 h, the culture plate was washed with PBS three times, and then 100 μl of DMEM containing 2 μg/ml trypsin (Gibco, United States) was added. After 48 h, the neutralization titer was calculated as the reciprocal of the highest dilution of serum that inhibited virus-specific CPE in duplicate wells (Lee et al., 2017).

### Statistical Analysis

All values are expressed as means ± standard deviations (SD) of the means. The statistical significance of all differences was evaluated with Student’s *t-*test in GraphPad Prism software version 5.0 (GraphPad Prism Inc., United States).

## Results

### Virus Isolation and *in vitro* Characterization

**FIGURE 1:**
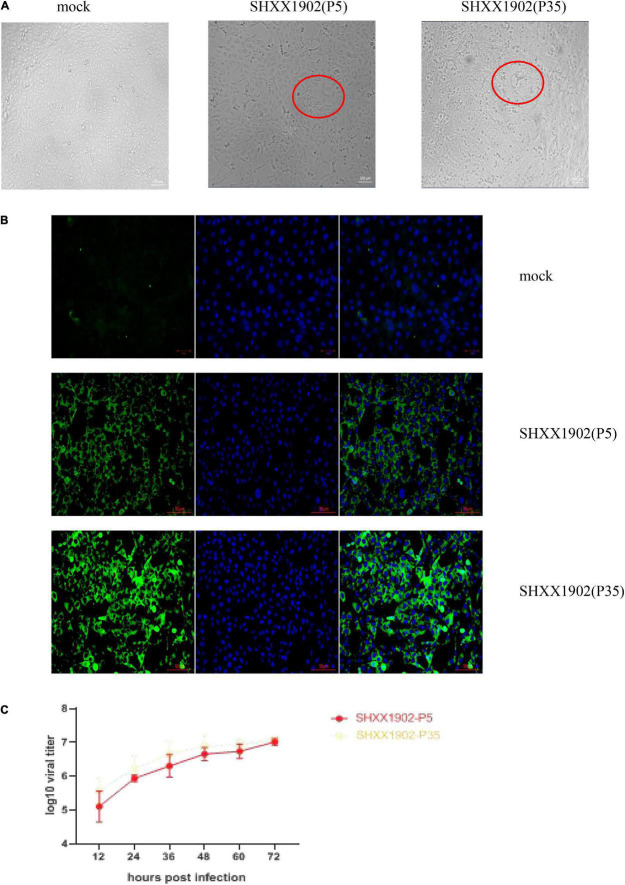
Cytopathology and growth properties of PEDV SHXX1902. **(A)** CPE formation in Vero cells infected with strain SHXX1902-P5 or SHXX1902-P35. PEDV-specific CPE was observed daily, and the cells were photographed at 24 hpi using an inverted microscope at a magnification of ×200. The red circle indicates the CPE. **(B)** For immunostaining, infected cells were fixed at 24 hpi and incubated with a monoclonal antibody directed against the S protein and then with Alexa-green-conjugated goat anti-mouse secondary antibody. The cells were then examined under a fluorescence microscope at ×200 magnification. **(C)** One-step growth kinetics of SHXX1902-P5 and SHXX1902-P35. At the indicated time points post-infection, the culture supernatants were harvested from SHXX1902-P5-infected Vero cells, and the viral titers were determined. There was no significant difference between the titers of the two passages (*P* > 0.05). CPE, cytopathic effect; PEDV, porcine epidemic diarrhea virus; DAPI, 4,6-diamidino-2-phenylindole; TCID50, median tissue culture infectious dose.

Porcine epidemic diarrhea virus strains SHXX1902-P5 and SHXX1902-P35 induced cytopathic effects (CPEs) typical of PEDV infection, such as cell fusion, syncytium formation, and Vero cell detachment. The detection of PEDV antigens with immunofluorescence assay (IFA) using an anti-PEDV spike (S)-specific monoclonal antibody confirmed viral propagation. The cytoplasm displayed the distinct staining pattern associated with syncytium formation. Neither CPE nor S-specific monoclonal antibody staining in the mock-infected cells was detected. Figure 1 shows CPE and corresponding IFA images in cells infected with passage P5 or P35. The SHXX1902-P5 and SHXX1902-P35 strains’ titers were determined. SHXX1902-P5 titers ranged from 10^5.11^ to 10^7.01^ TCID_50_/ml, while SHXX1902-P35 titers ranged from 10^5.58^ to 10^7.09^ TCID_50_/ml. There was no significant difference between the titers of the two passages (*P* > 0.05).

### Comparative Virulence of Subcultured Strains

Each group (groups 1–5) of five piglets was challenged orally with 10-fold serially diluted (10^–3^–10^–7^) SHXX1902-P5 virus, whereas the control animals in group 6 were administered a cell culture medium. All five piglets in group 1 (infected with viral dilution 10^–3^) developed watery diarrhea (score 3) by 3 dpi, which persisted throughout the study. Three, three, and one piglet in groups 2, 3, and 4, respectively, infected with viral dilutions 10^–4^–10^–6^, developed moderate-to-watery diarrhea (scores 2–3) by 3 dpi, which persisted throughout the study period (Table 1). In contrast, none of the five pigs in group 5 (infected with viral dilution 10^–7^) and those in the negative control group remained active and clinically unaffected throughout the 5-day experimental period. By 5 dpi, 100% of the piglets in group 1, 80% of those in group 2, 40% in group 3, and 20% of those in group 4 had died from PEDV-related clinical signs. The PDD_50_ and LD_50_ values for SHXX1902-P5 were determined indirectly and corresponded to theoretical cell culture infectious titers of 10^2.0^ and 10^2.84^ TCID_50_/ml, respectively.

**TABLE 1:** Summary of pig groups, group numbers, inocula, and pig diarrhea and death outcomes after inoculation.

Pig group	Pig numbers	Inoculum dilution*	Calculated inoculum infectious titers (log10 TCID_50_/mL)^†^	Diarrhea (percent)	Death (percent)
Group 1	5	10^–3^	4	5/5 (100)	5/5 (100)
Group 2	5	10^–4^	3	3/5 (60)	4/5 (80)
Group 3	5	10^–5^	2	3/5 (60)	2/5 (40)
Group 4	5	10^–6^	1	1/5 (20)	1/5 (20)
Group 5	5	10^–7^	0	0/5 (0)	0/5 (0)
Group 6	5	Cell culture media	−	0/5 (0)	0/5 (0)

*Each pig was inoculated orally with 1 mL of inoculum. ^†^Titers were calculated based on the titer of the original viral pool and the fold dilution.

### Pathogenicity of SHXX1902 Virus in Neonatal Piglets

Animal infection experiments were performed to examine the changes in the *in vivo* viral phenotype associated with the serial *in vitro* passage of strain SHXX1902. The pathogenicity of SHXX1902-P5 and SHXX1902-P35 was characterized in piglets. Twelve piglets were divided into three groups of four animals, each was challenged orally with SHXX1902-P5 (group 1) or SHXX1902-P35 (group 2), and the remaining piglets in the control group received cell culture medium. The clinical signs were recorded daily, and fecal swabs were collected pre- and post-challenge for the duration of the study. During the acclimation period, all the piglets were active, showed no clinical symptoms, and had normal fecal consistency, and their feces contained no PEDV genetic material. After the challenge, none of the negative control piglets developed clinical presentations typical of PEDV throughout the study.

**FIGURE 2:**
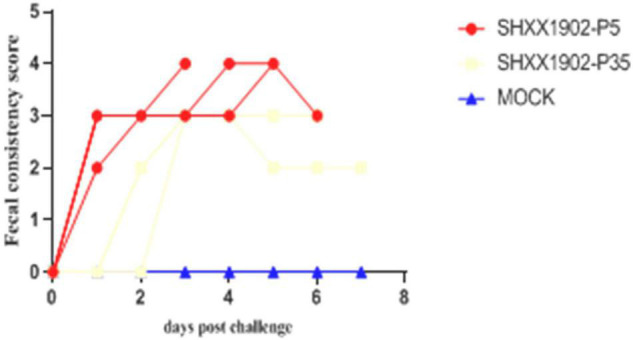
Fecal consistency scores in piglets inoculated with PEDV SHXX1902-P5, SHXX1902-P35, or mock inoculated by 7 dpi. {0 = normal and no diarrhea [mean cycle threshold (*Ct*) values of >45]; 1 = mild and fluidic feces; 2 = moderate watery diarrhea; 3 = severe watery and projectile diarrhea [mean *Ct* values of <20]; and 4 = death}.

In contrast, the SHXX1902-P5-challenged piglets (group 1) displayed clinical signs, including loss of appetite and diarrheic feces, by 1 dpi and suffered lethal watery diarrhea with vomiting after that. One pig had diarrhea [fecal consistency (FC) score = 2], and the other three pigs had watery diarrhea (FC score = 3) by 1 dpi, which all persisted for 1–7 days. However, no piglets in the SHXX1902-P35-challenged group displayed clinical signs, such as loss of appetite or diarrheic feces, at 1 dpi. Only two of the four SHXX1902-P35-challenged piglets (group 2) displayed clinical signs, including loss of appetite and diarrheic feces, by 2 dpi, or suffered lethal watery diarrhea with vomiting after that (Figure 2).

**FIGURE 3:**
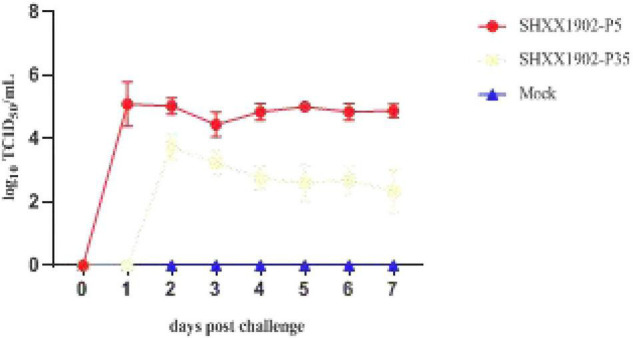
Viral shedding in piglets inoculated with PEDV SHXX1902-P5, SHXX1902-P35, or mock inoculated. PEDV titers in rectal swab samples at each time point were determined with RT-qPCR. The viral titers (log10 TCID_50_/ml) are the mean viral titers of all the piglets in each group, and error bars represent SD.

By 1 dpi, all the animals in group 1 were positive for PEDV, as determined with RT-PCR, with mean *Ct* values of 15.78–19.33 (equivalent to 10^4.07^–10^5.77^ TCID_50_/ml), and shed significantly smaller amounts of PEDV in their feces with *Ct* values ranging from 20.80 to 22.35 (10^4.57^–10^5.14^ TCID_50_/ml) until 7 dpi (Figure 3). No fecal shedding of PEDV was detected in any of the five piglets in group 2 at 1 dpi. PEDV was detected in only three piglets in group 2 at 2 dpi, with mean *Ct* values of 18.09–20.59 (10^3.11^–10^4.03^ TCID_50_/ml), which decreased to the lowest level at 7 dpi. Overall, the quantities of virus in the feces of the group 2 animals were significantly lower than those in group 1, with a wide *Ct* range of 24.46–28.34 (10^1.50^–10^3.16^ TCID_50_/ml) until the termination of the study, indicating the limited viral shedding (an almost 3-log reduction) of strain SHXX1902-P35. The negative control piglets remained active throughout the experimental period, with neither diarrhea nor fecal PEDV shedding.

**FIGURE 4:**
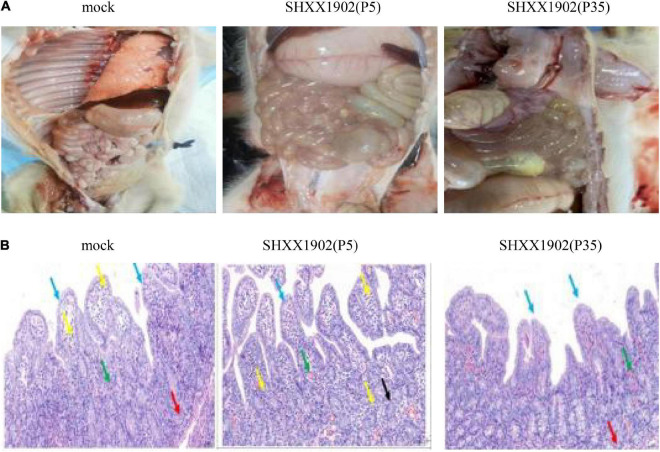
Histopathological analysis. **(A)** Comparison of small intestinal lesions in different groups (mock, P5, and P35) at necropsy at 3 dpi. **(B)** Hematoxylin-and-eosin-stained jejunum tissue sections from the different groups at 3 dpi.

At 3 dpi, two piglets from the three groups were necropsied. Neither macroscopic nor microscopic intestinal lesions were evident in the negative control piglets. The virulent SHXX1902-P5-infected piglets showed typical macroscopic gross PED-like lesions. Their small intestines were dilated with accumulated yellowish fecal fluid and had thin transparent walls resulting from villous atrophy, whereas their other internal organs appeared normal. In contrast, all the animals infected with SHXX1902-P35 (group 2) displayed markedly visible pathological lesions in their gastrointestinal tracts, albeit with normal bowel wall thickness, similar to that in the negative control group. Altogether, compared with strain SHXX1902-P5, SHXX1902-P35 showed weakened virulence with an attenuated phenotype in highly susceptible piglets under experimental conditions (Figure 4A). The microscopic assessment revealed the following: MOCK: The mucosa and submucosa bulged into the intestinal cavity to form more folds (black arrow); the lamina propria bulged into the intestinal cavity to form more intestinal villus structures (blue arrow); the intestinal villi were underdeveloped, differed in height, and were fat or thin, and the mucosal epithelium was complete. Some capillaries in the lamina propria were slightly congested (green arrow), and a small amount of lymphocyte infiltration was seen on top of a small number of intestinal villi (yellow arrow); the glands in the lamina propria were evenly distributed and closely arranged, and Pan’s cells at the bottom had disappeared (red arrow). SHXX1902-P5: The lamina propria bulged into the intestinal cavity to form many intestinal villi (blue arrow). The intestinal villi were evenly distributed and long, and the mucosal epithelium was complete. A large number of capillaries in the lamina propria were congested and dilated (green arrow), and more lymphocyte infiltration was seen (yellow arrow); the glands of the lamina propria were loosely arranged, some were irregular in shape (black arrow), and Pan’s cells at the bottom had disappeared. SHXX1902-P35: The mucosa and submucosa bulged into the intestinal cavity to form a small number of folds (black arrow), and the lamina propria bulged into the intestinal cavity to form a large number of intestinal villi (blue arrow). The intestinal villi were underdeveloped, differed in height, and were fat or thin, and the mucosal epithelium was complete. Some capillaries of the lamina propria were slightly congested (green arrow); the glands of the lamina propria were evenly distributed and closely arranged, and Pan’s cells at the bottom had disappeared (red arrow); no obvious inflammatory cell infiltration was observed (Figure 4B).

### Protective Efficacy of the Inactivated SHXX1902-P5 Vaccine

To evaluate the protective efficacy of the inactivated SHXX1902-P5 strain, we used an intramuscular homologous prime-boost vaccination scheme, administered 2 weeks apart before farrowing. At 5 and 2 weeks pre-partum, the three sows in group 1 were primed and boosted with the AJ1102 vaccine, and the three sows in group 2 were orally primed and boosted with the inactivated SHXX1902-P5 strain. None of the sows in the unvaccinated group had any adverse reactions. At farrowing, there were no significant differences in the reproductive performance of the vaccinated and unvaccinated sows.

**FIGURE 5:**
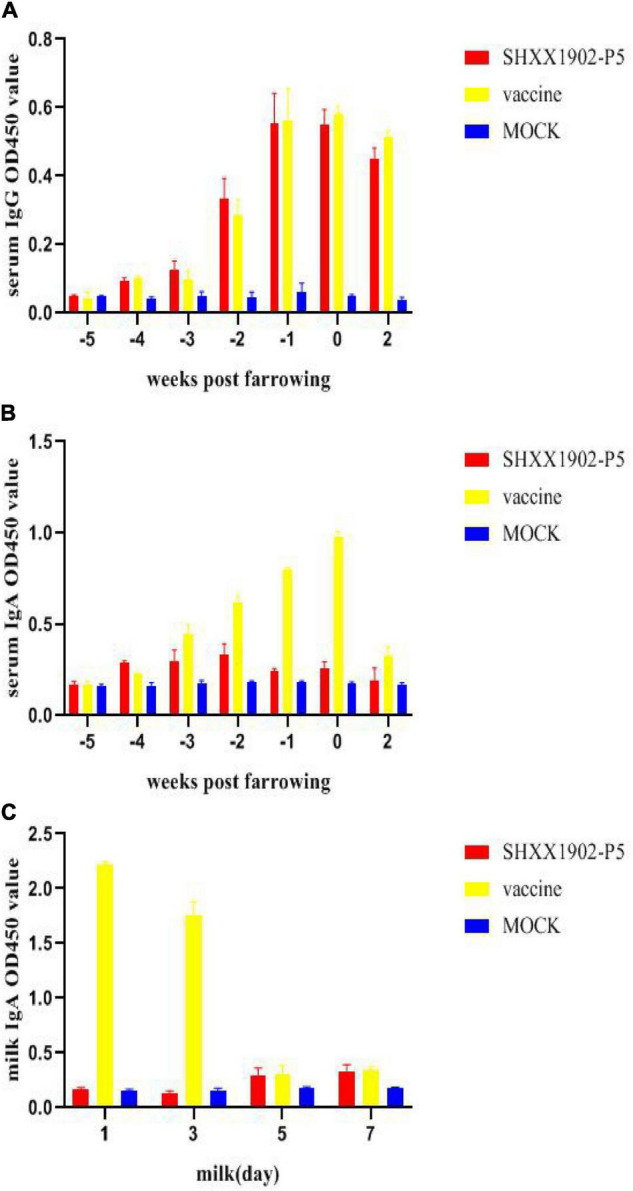
Levels of PEDV-specific IgG and IgA antibodies in the serum and IgA antibodies in the milk of sows after immunization with different strains. Levels of PEDV-specific IgG antibodies in the serum **(A)**, IgA antibodies in the serum **(B)**, and IgA antibodies in the milk **(C)** were determined with ELISAs. All data are shown as means ± SD.

After the first immunization of sows, the serum IgG levels induced by SHXX1902-P5 and AJ1102 were similar. After the second immunization, the serum IgG titers increased (Figure 5A). However, the IgA levels induced by AJ1102 were higher than those induced by SHXX1902-P5 after the second immunization (Figure 5B). The IgA levels in the milk from the AJ1102-vaccinated sows were higher than those from the SHXX1902-P5-vaccinated sows on days 1 and 3. However, the IgA levels induced by SHXX1902-P5 and AJ1102 were similar on days 5 and 7 (Figure 5C).

**FIGURE 6:**
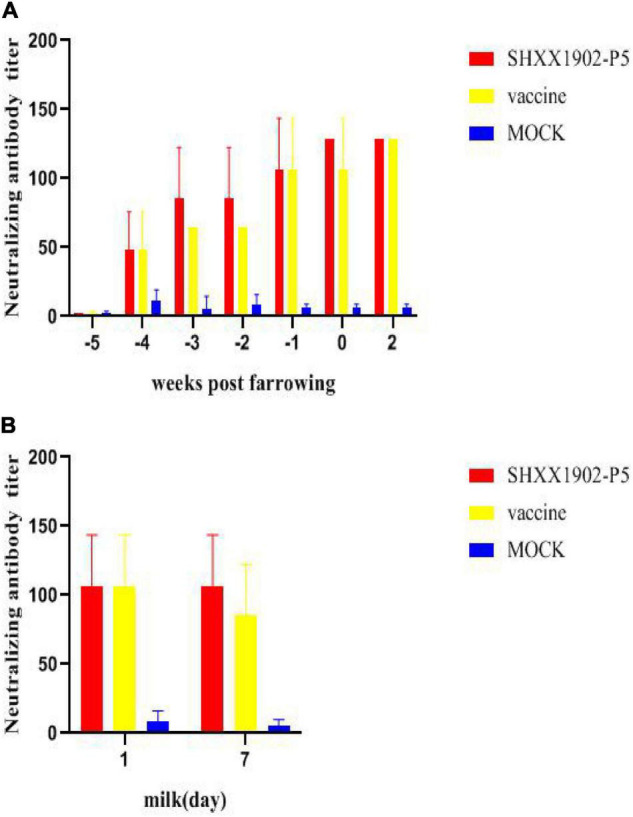
Levels of neutralizing antibodies in the serum and the milk of sows after immunization with different strains. Levels of PEDV-specific neutralizing antibodies in the serum **(A)** and PEDV-specific neutralizing antibodies in the milk **(B)** were determined. All data are shown as means ± SD.

The results of PEDV-neutralizing antibody tests showed that the neutralizing antibody levels of the two immunized groups gradually increased until 42 days after the first immunization. The neutralizing antibody titers in serum samples of the two immunized groups were comparable (*P* > 0.05, Figure 6A). There were no significant difference in the neutralizing antibody titer in the colostrum and the milk on the 7th day after farrowing between the two immunized groups (*P* > 0.05, Figure 6B).

## Discussion

With the pandemic of the PEDV G2b strain ravaging the global swine industry, many novel G2b vaccines have been developed in China. Since 2010, G2c PEDV has spread throughout the world. Many novel G2b vaccines cannot provide sufficient protection against G2c PEDV strains in China on many pig farms. We first isolated the PEDV G2c field strain SHXX1902, which can be efficiently propagated in a Vero cell suspension to address this problem. The G2c isolate SHXX1902-P35 displayed similar growth characteristics to SHXX1902-P5 in terms of its cytopathology, infectious titer, and replication kinetics in Vero cells, as reported previously [9]. With whole-genome analyses, several of the strains circulating in China since 2015 (including XM2-4, CH/HNAY/2015, CH-HB2-2018, and JSCZ1601) have been shown to belong to the same subgroup (G2c) as SHXX1902-P5. However, the S gene of SHXX1902-P5 belongs to subgenotype G2a, whereas the whole genome of SHXX1902-P35 belongs to G2b. It has been reported that most viral mutations occur during extensive passaging of viruses in cell culture, and these mutations have been correlated with viral attenuation. Therefore, we compared the pathogenicity of the two isolates, SHXX1902-P5 and SHXX1902-P35.

Strain SHXX1902-P5 caused earlier viral shedding (at 1 dpi), higher titers of shedding virus, more severe macroscopic and microscopic symptoms, and a higher death rate than strain SHXX1902-P35. However, SHXX1902-P35 still caused mild PEDV syndrome. Therefore, SHXX1902-P35 requires further attenuation before its preparation as an attenuated vaccine. Noticeably, strain SHXX1902-P35 had changed into the G2b subtype. Therefore, to prevent G2c PEDV infection, we selected SHXX1902-P5 and constructed an inactivated vaccine based on the P5 virus.

Paudel et al. (2014) reported that PEDV-negative sows were given one of four vaccine treatments: no vaccination (control group); vaccination with a killed virus, with a killed virus booster (K/K); vaccination with a live virus, with a live virus booster (L/L); and vaccination with a live virus, with a killed virus booster (L/K). All the sows were vaccinated intramuscularly twice, at 5 and 3 weeks before farrowing. The group treated with the K/K strategy had the highest IgG, IgA, and neutralizing antibodies in their sow sera, colostrum, and piglet sera, with the lowest levels in the L/L group. However, in our study, the IgA titer after the L/K treatment was much higher than that in the K/K colostrum and milk on day 3 after farrowing. As the second immunization of AJ1102 was an attenuated vaccine, it can induce mucosal immunity.

In the first reported experimental study of a US PEDV isolate in sows, four sows from a herd naturally infected with a mild SINDEL strain of PEDV were used. As we all know, G2c PEDV is also called the “SINDEL” strain. At about 3 days of age, their piglets were orally challenged with the virulent virus. All the piglets from the PEDV-positive sows survived this challenge, whereas those from the PEDV-naive sows experienced a 33% mortality rate (Goede et al., 2015). That study demonstrated that the SINDEL strain of PEDV could produce lactogenic immunity and provide the direction of oral vaccine using our isolated strain. Lactogenic immunity is described as the continuous supply of passively acquired immunoglobulins through the ingestion of colostrum and milk. IgG is dominant in colostrum and is transudate from sow serum for piglet protection. SIgA is dominant in milk (Langel et al., 2016). In our study, the IgG titers were similar in the AJ1102- and SHXX1902-P5-vaccinated sows, from the first day after vaccination to 14 days after farrowing. Therefore, our strain is also a promising vaccine candidate against SINDEL strains.

Porcine epidemic diarrhea virus mainly infects the intestines of young piglets, with transient viremia in the serum (Jung et al., 2014). Therefore, mucosal immunity in neonates at birth and throughout the nursing period is important. Suckling piglets *via* colostrum and milk is crucial for their immediate protection against PEDV. It has been reported that the protection level is associated with the antibodies in the milk, but not those in the serum, of the sows (Bohl et al., 1972; Saif et al., 1972; Bohl and Saif, 1975). The injection of an inactivated vaccine or organization of own vaccine induces the non-mucosal immune pathway and does not produce secretory IgA (sIgA). The injection of a live or a purified live virus entails the same problems as the injection of an inactivated vaccine. Oral live vaccines are greatly affected by pepsin, and this effect may vary across strains. The intramuscular injection only improves the single-chain variable fragment (scFv) level in the colostrum and does not induce sIgA. The existence of the blood–milk barrier means that scFv can only neutralize some free viruses in the colostrum stage. In the present study, the IgA titer declined rapidly in the milk immunized with AJ1102 by day 5 after farrowing, which is consistent with the existence of the blood–milk barrier. Bohl and Saif (1975) discovered the gut–mammary–sIgA axis (Langel et al., 2016), explaining why sows naturally infected or orally infected with live TGEV and recovered from the virus had persistently high levels of sIgA antibodies in their milk, which protected their piglets from TGEV infection. Therefore, the induction of the mucosal immune pathway is a promising strategy. However, the neutralizing antibody titer of serum can reach 1:128 after the farrowing day in all immunized sows. The neutralizing antibody titer of colostrum and milk at day 7 was 1:85 or 1:107. As a result, our isolated strain was a promising candidate strain.

## Data Availability Statement

The original contributions presented in the study are included in the article/supplementary material, further inquiries can be directed to the corresponding author.

## Ethics Statement

The animal study was reviewed and approved by Shanghai Animal Diseases Prevention and Control Center.

## Author Contributions

F-FG: conceptualization and writing—original draft preparation. H-XS, D-QY, and XL: methodology. H-BJ, JW, and H-JZ: formal analysis and investigation. F-FG and L-SK: writing—review and editing. All authors contributed to the article and approved the submitted version.

## Conflict of Interest

The authors declare that the research was conducted in the absence of any commercial or financial relationships that could be construed as a potential conflict of interest.

## Publisher’s Note

All claims expressed in this article are solely those of the authors and do not necessarily represent those of their affiliated organizations, or those of the publisher, the editors and the reviewers. Any product that may be evaluated in this article, or claim that may be made by its manufacturer, is not guaranteed or endorsed by the publisher.
